# Emergency department use of a high-sensitivity point-of-care troponin assay reduces length of stay: an implementation study preliminary report

**DOI:** 10.1093/ehjacc/zuae114

**Published:** 2024-10-15

**Authors:** John W Pickering, Laura R Joyce, Christopher M Florkowski, Vanessa Buchan, Laura Hamill, Martin P Than

**Affiliations:** Department of Medicine, University of Otago Christchurch, Christchurch, New Zealand; Emergency Department, Christchurch Hospital, Private Bag 4710, Christchurch 8140, New Zealand; Department of Surgery and Critical Care, University of Otago Christchurch, Christchurch, New Zealand; Canterbury Health Laboratories, Christchurch Hospital, Christchurch, New Zealand; Canterbury Health Laboratories, Christchurch Hospital, Christchurch, New Zealand; 24hr Surgery, Pegasus Health (Charitable), 401 Madras Street, Christchurch, New Zealand; Department of Medicine, University of Otago Christchurch, Christchurch, New Zealand; Emergency Department, Christchurch Hospital, Private Bag 4710, Christchurch 8140, New Zealand; Department of Emergency Medicine, University of Kansas Medical Center, 4000 Cambridge Street Mailstop 1019, Kansas City, KS 66160, USA

**Keywords:** High-sensitivity cardiac troponin, Point of care, Acute myocardial infarction, Emergency department, Emergency room, Accelerated diagnostic pathway

## Abstract

**Aims:**

Point-of-care (POC) high-sensitivity troponin (hs-cTn) assays within a clinical pathway may safely reduce length of stay (LoS) for patients presenting to the emergency department (ED) with possible acute myocardial infarction (AMI). In this early report, we present the first evaluation of a POC hs-cTn in real-life care.

**Methods and results:**

In adult patients presenting to ED investigated for possible AMI, we compared the LoS in patients assessed with a troponin in the 8 weeks before (usual-care phase) and the 8 weeks following introduction of the Siemens Atellica VTLi POC hs-cTnI for decision-making (intervention phase). The VTLi replaced the laboratory (Beckman Coulter) assay as the default hs-cTn test within the clinical pathway. This was the only change to the pathway process. The safety outcome was first event AMI or cardiac death within 30 days. There were 2376 presentations in the usual-care phase with 188 individuals with AMI and 2392 in the intervention phase with 198 AMI. In the intervention phase, there was a mean (95% CI) reduction in LoS of 32 min (22–41 min) compared with the usual-care phase. This represents 21.4 fewer patient-hours in the ED each day (1196 in the 8-week period). In both phases, the pathway correctly identified all cases of AMI at index attendance. There were four follow-up events (two usual-care, two intervention) within 30 days.

**Conclusion:**

The deployment of a hs-cTn POC analyser into a large ED safely reduced length of stay. If translatable to other EDs, this could represent an important advancement to patient care.

**Trial registration:**

Australia New Zealand Clinical Trials Registry, No. ACTRN12619001189112.

**Key points**

- First use of a high-sensitivity troponin (hs-cTn) point-of-care (POC) assay within a structured accelerated diagnostic pathway for acute coronary syndrome substantially reduced emergency department length of stay compared to a laboratory hs-cTn assay.
- The safety with the POC hs-cTn assay was equivalent to that of a laboratory hs-cTn assay.
- The impact on change in length of stay within alternative pathways and settings remains to be determined.

## Introduction

Emergency departments (EDs) struggle with high patient numbers and crowding. High-sensitivity troponin assays (hs-cTn) facilitate more rapid assessment of possible acute coronary syndrome (ACS) through use of accelerated diagnostic pathways (ADPs) that include risk stratification to low-risk after one blood sample. The turn-around time from blood draw to results availability to the clinician can delay early decision-making and potential discharge. When results become available, clinicians are often unable to act immediately because they are busy with other patients. Point-of-care (POC) hs-cTn has been proposed to expedite decision-making and reduce ED length of stay (LoS).^1,2^ Here, we report, pre-planned, early ‘before and after’ findings from first use within real-life clinical practice of a POC hs-cTn analyser, the Siemens (Erlangen, Germany) Atellica VTLi hs-cTnI.^3–7^ The VTLi has an 8 min turn-around time and regulatory approval with European conformité européenne (CE) marking.

## Methods

New Zealand (NZ) EDs use ADPs comprising hs-cTn, electrocardiogram, and risk stratification based on patient history and symptoms.^8,9^ Some patients with no new ischaemia on ECG, low-risk history and symptoms, and a first troponin concentration measured at least 3 h following symptom onset and less than a low-risk threshold are eligible for discharge home. Others will have a second troponin measurement, normally 2 h after the first, after which a disposition decision is made.

Improving Care by FAster risk-STratification in the EmeRgency department (ICare-FASTER) is a quality improvement initiative where the VTLi is introduced into multiple NZ EDs in a stepped wedge fashion.^10^ Christchurch Hospital ED, approximate annual attendance 140 000, is the sentinel site where implementation challenges can be addressed and an initial assessment of the VLTi pathway made.^10^ A run-in period was pre-planned where the VTLi is first in use for clinical decision-making. This ‘familiarization’ period is not part of the primary analysis because time is needed to allow staff to become skilled in the use and application of the test. This report compares LoS, and 30-day outcomes, in patients assessed with a troponin in the eight weeks before (usual-care phase) and the eight weeks following the introduction of the VTLi for decision-making (intervention phase). Differences between study arms are reported as the measured difference with a 95% confidence interval.

Eligible patients were adults (≥18 years) presenting to the ED in whom attending clinical staff ordered cardiac troponin test(s) because there was a perceived need to investigate for possible AMI alone or as part of a differential diagnosis. The usual-care phase was from 6 June 2023 to 31 July 2023 where troponin was measured by the laboratory (Beckman) assay, which was the only test used for decision-making. The intervention phase was from 1 August 2023 to 25 September 2023 where troponin could be measured by the POC (VTLi) assay, the default test for risk stratification and discharge decision-making. The VTLi test was performed on whole blood by ED nurses (*n* = 260) trained to perform the test. Both phases incorporated a venous blood draw into a lithium heparin tube. The use of the VTLi assay instead of the Beckman assay was the only change to the ADP (see Supplementary material online, *Figure S1*). The use of the Beckman assay during the intervention phase was expected and acceptable in certain circumstances, such as an invalid result on the VTLi device or patients who had recent results via the Beckman assay.^10^ In cases where results from both assays were available on the same sample, clinicians were instructed to act on the worse result (safety first). Once a decision to admit was made, then all inpatient decisions were made based upon the concentrations of the usual Beckman assay. The VTLi hs-cTnI assay utilizes secondary cTnI antibodies directed at epitopes 23–29, 87–91, and an anti-cTnC antibody.^4^ Assay characteristics are described in *Table 1*.

**Table 1:** Characteristics of troponin assays used

Assay	LoB	LoD	Concentration at 20% CV	Concentration at 10% CV	99th centile of the URL (plasma)	Reportable measuring range
Siemens Atellica® VTLi POC hs-cTnI^12^ (ng/L)	0.55	1.2 plasma, 1.6 WB	2.1 plasma, 3.7 WB	6.7 plasma, 8.9 WB	18 (F) 27 (M)	1.2–1250
Beckman Coulter Access hs-cTnI^13^ (ng/L)	1.7	2.3	2.3	5.6	>10 (F) > 20 (M)	2.3–27 027
Assessment pathway group definitions: single test risk stratification for possible early discharge requirements Eligible for discharge if all of (a) to (e) are true. See also the Supplementary material online, *Figure S1*.						
Siemens Atellica® VTLi POC hs-cTnI assay	(a) hs-cTnI < 7 ng/L, (b) Normal/unchanged ECG, (c) EDACS < 21, (d) No red flags, and (e) Symptom onset ≥3 h pre-test.					
Beckman Coulter Access hs-cTnI assay	(a) hs-cTnI < 6 ng/L, (b) Normal/unchanged ECG, (c) EDACS < 21, (d) No red flags, and (e) Symptom onset ≥3 h pre-test blood draw.					

LoB, level of blank; LoD, level of detection; CV, coefficient of variation; URL, upper reference limit; POC, point of care; F, female; M, male; WB, whole blood; ECG, electrocardiogram; EDACS, Emergency Department Assessment of Chest pain Score.

The single-test low-risk threshold of the VTLi, used in conjunction with an electrocardiogram and the Emergency Department Assessment of Chest pain Score (EDACS), was 7 ng/L (<7 was negative).^6^ Participants were identified from the ED electronic attendance record. The primary outcome was ED LoS (from ED arrival until either discharged home or admitted to an inpatient ward). Patients were electronically followed-up for 30 days using their National Health Index identifier (NHI). The NHI is a unique identifier of every individual who uses NZ health services enabling complete follow-up. The safety metric was first event acute myocardial infarction (AMI) or cardiac death following the initial presentation in each phase for each patient. ICD-10 codes identified AMI. Patients discharged with a troponin result below the low-risk threshold were adjudicated for potential missed cases by at least two experienced physicians using the 4th Universal Definition for myocardial infarction.^11^ The Southern Health and Disability Ethics Committee (21/STH/9) determined that no individual consent was required.

## Results

During the 16-week study period, there were 4768 presentations with a troponin measurement (2376 presentations usual-care; 2392 intervention). The mean (SD) age was 62.0 years (18.5 years), and 49.7% were female. The mean age and proportion female were almost identical in the two phases. In the intervention phase, 1616 (67.6%) were assessed with the VTLi of whom 261 also had a Beckman hs-cTnI measured. The other 774 (32.3%) were assessed by the Beckman only.

**Figure 1:**
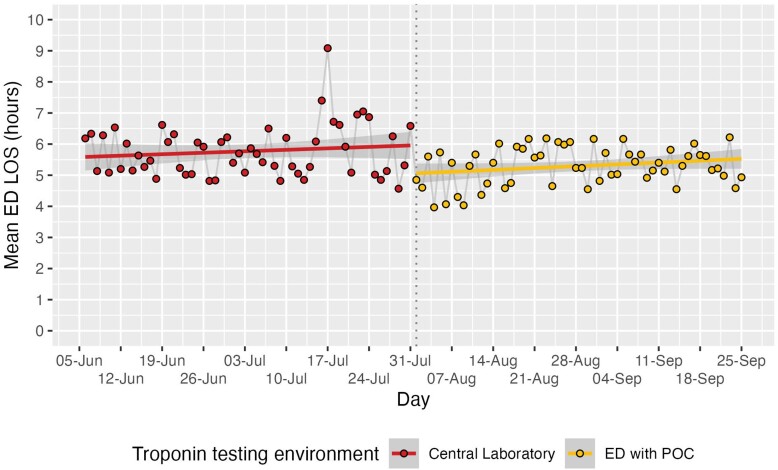
Daily mean lengths of ED stay of patients receiving a troponin test for the eight weeks before and after the deployment of the POC test in the ED.

The median ED LoS fell from 313 min (usual-care) to 286 min (intervention), *Table 2*. The mean LoS of all patients having a troponin measured also fell, *Figure 1*, resulting in a mean (95% CI) reduction of 32 min (22–41 min). This represents 21.4 fewer patient-hours in the ED each day (1196 h in the 8-week period). The proportion of patients discharged within 2 h nearly doubled from 2.7% to 5.2%. The mean reduction in turn-around time was 59.3 min (*Table 2*), longer than the mean reduction in ED LoS, reflecting that additional factors influence LoS. A detailed time in motion study would be needed to identify these and possible other ways to reduce LoS.

**Table 2:** Results

Metric	Usual-care phase (*n* = 2376)	Intervention phase (*n* = 2392)	Difference (95% CI)
Mean ED LoS (SD): minutes	350 (181)	319 (163)	−31.7 (−42.0 to −22.0)
Median ED LoS (IQR): minutes	313 (227–431)	286 (210–390)	−28.2 (−39.1 to −19.0)
ED LoS < 2 h % (*n*)	2.7% (65)	5.3% (126)	2.6% (1.4–3.6%)
ED LoS < 3 h % (*n*)	13.1% (311)	17.1% (410)	4.0% (2.1–6.0%)
Cumulative time in ED (h)	13 860	12 717	−1143 (−2072 to −300)
Admitted as an inpatient % (*n*)	40.2% (955)	41.0% (981)	0.8% (−2.1–3.4%)
Index admission AMI^a^ (per individual)	8.5% (188)	8.9% (198)	0.4% (−1.2–2.0%)
Mean (IQR) time from blood draw to posting of results: minutes	128 (216)	68 (175)	−59.3 (−70.4 to −47.7)
Median (IQR) time from blood draw to posting of results: minutes	71 (56–109)	12 (11–63)	−59.6 (−61.0 to −58.0)
Median (IQR) time from blood draw to posting of results with the VTLi only: minutes		11 (11–12)	
Patients not admitted			
Mean ED LoS (SD): minutes	320 (156)	286 (144)	−33.5 (−22.7 to −44.2)
Median ED LoS (IQR): minutes	285 (215–383)	258 (192–348)	−27.3 (−15.0 to −41.0)
Patients assessed only with the VTLi assay			
Mean ED LoS (SD): minutes		299 (154)	
Median ED LoS (IQR): minutes		272 (197–363)	
Patients assessed with both assays			
Mean ED LoS (SD): minutes		326 (166)	
Median ED LoS (IQR): minutes		293 (207–404)	
Patients assessed only with the Beckman assay			
Mean ED LoS (SD): minutes		350 (172)	
Median ED LoS (IQR): minutes		313 (232–436)	

ED LoS, emergency department length of stay; AMI, acute myocardial infarction; SD, standard deviation; IQR, interquartile range.

^a^Identified by ICD10 codes I21.0, 21.1, 21.2, 21.3, 21.4, 21.9, 22.0, 22.1, 22.8, 22.9.

There were 188 individuals with AMI in the usual-care phase and 198 with AMI in the intervention phase. In both phases, the pathway correctly identified all cases of AMI at index attendance.

On follow-up, four patients, (two in each phase), had an AMI in the 30 days after completing the index visit assessment and being discharged home with peak Beckman hs-cTnI for the latter AMI event ranging from 113 to 214 ng/L.

In the usual-care phase, one patient with known multi-vessel coronary disease (not suitable for intervention procedures) and on maximal medical therapy had chronically raised troponin. One represented 9 days later with non-ST-elevation myocardial infarction (NSTEMI) that was managed conservatively. A second patient was discharged after inpatient-specialist advice and medication changes; she represented 21 days later with NSTEMI and started dual antiplatelet therapy.

During the intervention phase, one patient self-discharged from ED after diagnosis of AMI, who was managed medically on re-admission 3 days later. They were already awaiting cardiovascular multi-disciplinary planning review regarding management of known triple vessel disease. A second patient with acute foot ischaemia and gout had an incidental discovery of a type 2 AMI during a next-day representation for polyarthropathy and fever. They did not receive additional cardiac investigations or management.

There were 125 deaths [72 usual-care (3.2%), 53 intervention (2.4%), *P* = 0.09] within 30 days. Four died in ED, 113 following admission, and only 8 (4 usual-care, 4 intervention) after discharge from ED. None of these eight patients died of a cardiac cause; four died of a condition for which they were receiving palliative care, three of sepsis, and one of a cerebrovascular accident.

## Discussion and conclusion

This early and first report on the real-life deployment of a hs-cTn POC analyser into the clinical pathway at a busy tertiary ED immediately, and safely, reduced LoS. A study strength is its real-world deployment. However, it is not a randomized controlled study and the time of year of the phases differed slightly. A limitation was some use of coded diagnoses, however in NZ, coded outcomes for AMI correlate well with blinded adjudication (98% agreement).^8^ Nor can the results be assumed to apply to hospitals using other ADPs such as the ESC 0/2h algorithms.

If this study’s results are translatable to other EDs with alternative pathways, other central laboratory assays, and/or different baseline lengths of stay, this could represent an important advancement to patient care-delivery. We recommend further studies in other settings to help define the factors that determine utility of this assay.

## Supplementary Material

zuae114_Supplementary_Data

## Data Availability

The data underlying this article cannot be shared publicly due to ethics and Māori data sovereignty requirements and the possibility of identifying individuals.
