# Supplementary material for: Emergency department use of a high-sensitivity point-of-care troponin assay reduces length of stay: an implementation study preliminary report
Source: Eur Heart J Acute Cardiovasc Care. 2024 Oct 15;13(12):838–42. doi: 10.1093/ehjacc/zuae114 (PMC11666305; doi:10.1093/ehjacc/zuae114)
Supplement: zuae114_Supplementary_Data [file zuae114_supplementary_data.pdf]

A — STEP A - Assess for Myocardial Infarction and 30-day risk of cardiac event

For all patients being discharged, complete ***both*** STEP A and STEP B (pg2)

USE ONLY IF CARDIAC ISCHEMIA IS THE PRIMARY CONCERN (requiring TnI testing - not for myopericarditis, heart failure, arrythmias)
Do not use if inpatient admission or investigation for another potential diagnosis is required.
Include: Is suspected ischemic cardiac chest pain the MAIN presenting problem requiring investigation today **and** if it was excluded could the patient be discharged to outpatient follow-up? ⇨ use this pathway
Exclude: Can myocardial infarction be excluded based on history and examination only, without measuring TnI (e.g. clearly musculoskeletal)? ⇨ exit this pathway

Patients referred by GP to Cardiology ⇨ seen by Cardiology. ED team can initiate blood tests and resuscitate as required.
Patients self-presenting ⇨ seen by ED.

CONSIDER RED FLAGS

SCREEN FOR STEMI and consider alternative causes, e.g.	Screen for high-risk features of aortic dissection (see HealthPathways) and ACS Red Flags:	**Red flag(s) present = Not for low-risk pathway** Manage as clinically indicated
• Pulmonary embolism<br>• Pneumothorax<br>• Abdominal cause e.g., Pancreatitis<br>• Oesophageal rupture	• Ongoing chest pain thought likely to be ischaemic<br>• Haemodynamic instability<br>• History suggestive of crescendo angina (increasing severity or frequency)<br>• Ischaemic changes on ECG, not known to be old	

Stable patients (no red flags) with possible ACS as the only differential diagnosis requiring ED investigation now

Troponin results shown as: Troponin by Siemens POC assay *[troponin by Beckman central lab assay]*

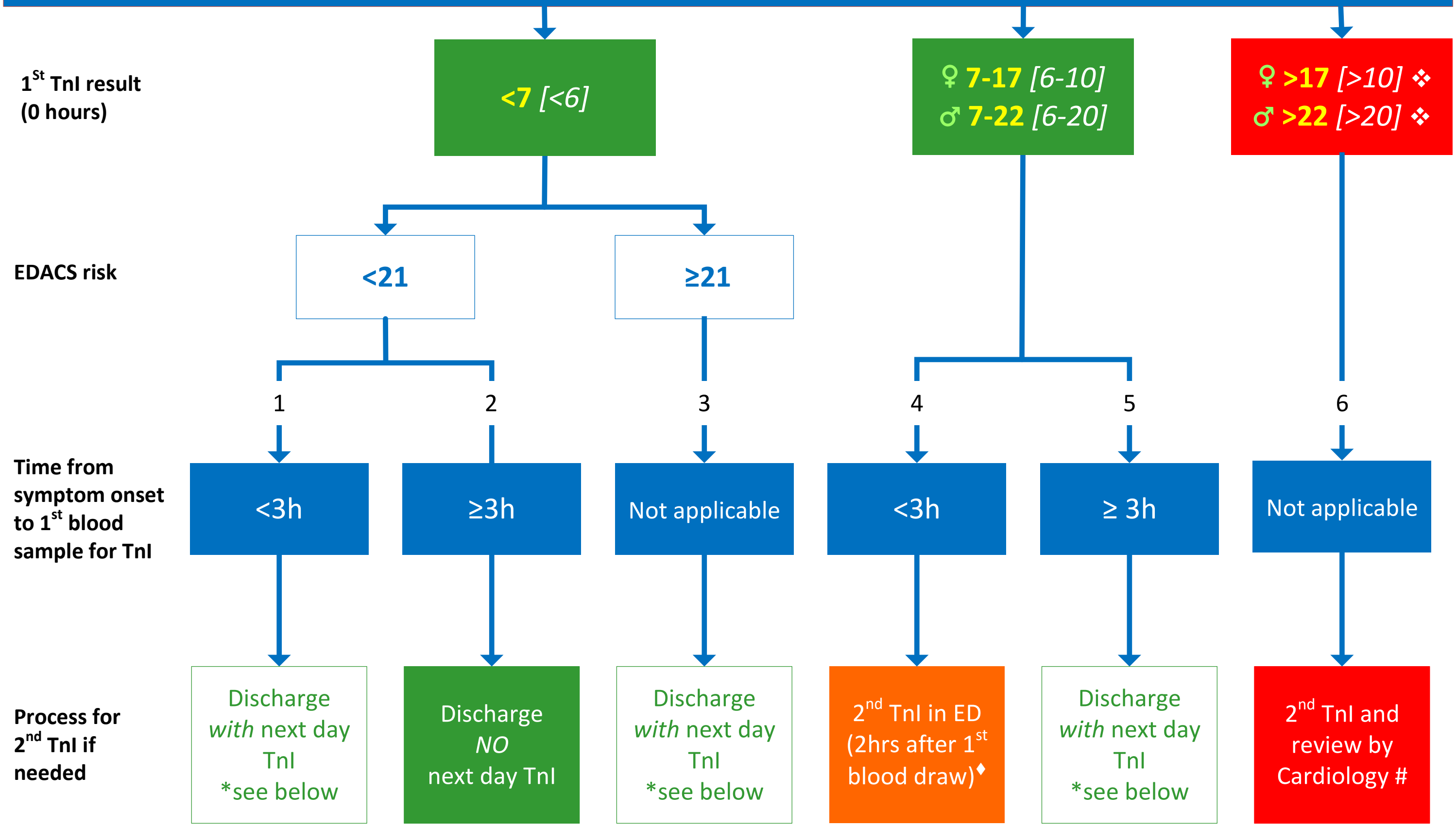

Now proceed to **Step B** on page 2

CTCA: CT coronary Angiogram **ETT:** Exercise Treadmill Test **TnI:** High-sensitivity cardiac troponin I

***Next day TnI via Acute Demand Nursing. It is *essential* to make e-referral and make telephone call to 0800 111 900.**
***Give patient blood test form and information sheet. (See Hospital HealthPathway – ACS for further guidance and printable forms)**
***Consider patient convenience and ability to attend when planning next day troponin (a repeat test in ED is a potential alternative).**
♦ If 2nd TnI positive, ♀ >17 *[>10]*, ♂ >22 *[>20]* or change between 1st and 2nd TnI samples ≥4 *[≥4]* patient should be discussed with Cardiology.
# Consider Gen Med referral for patients with frailty, comorbidity, or known CAD not suitable for invasive strategy.
❖ See guidance for chronic troponin elevation on following page
♀ Female ♂ Male

B — STEP B - Assess the need for secondary investigations for CAD (after AMI ruled out in Step A)

** Ruling out AMI does not exclude underlying CAD **

Troponin results shown as: Troponin by POC assay *[troponin by Beckman central lab assay]*

A — Chest pain is considered to be very low suspicion of CAD or non-cardiac (e.g. single short episode)
TnI **<7** *[<6]* (unless risk factors – see box C and D below)

→ YES → **ACTION**
Discharge to GP and politely request Cardiac risk profiling if not done within preceding 5 years

↓ NO *(therefore consider CAD)*

B — Marked frailty or comorbidity
OR
Known CAD not suitable for invasive strategy

→ YES → **ACTION**
Consider Cardiology SMO Virtual Review to optimise medical management (see below)[Ψ]
Otherwise follow-up by GP

↓ NO

C — **Risk factors requiring ED Cardiology consult:**

- TnI ♀ **>17** *[>10]* or ♂ **>22** *[>20]* ❖
- new ischaemia on ECG
- ongoing pain (if ED concerned it might be ischaemic)
- concerns with follow-up etc

→ YES → **ACTION**
Refer to Cardiology reg in ED
Direct discussion between senior Cardiology and ED doctor may help decision-making
All such patients not seen by Cardiology Reg in ED must be referred for Cardiology SMO virtual review

↓ NO

D — **Risk factors needing outpatient tests and Cardiology review:**

- High normal TnI ♀ **7-17** *[6-10]* or ♂ **7-22** *[6-20]*
- Recurrent symptoms not recently investigated
- High risk patients such as known history of CAD, or people with high exposure to multiple risk factors (e.g. Māori)

→ YES → **ACTION: either of**
A) Refer directly for ETT (see below)[♦] **UNLESS** patient:
- has abnormal resting ECG such as LBBB or AF
- had recent 'normal' investigations (see below)[ǂ]
- is not suitable for ETT (eg physically unable, or more specific tests are required, eg CTCA, invasive angiogram or echo)

OR
B) Refer to Cardiology SMO Virtual Review[Ψ] if not suitable for ETT

Dynamic TnI change (delta △): A significant (+ve) △ is considered present at **≥4** *[≥4]*ng/L (up to 20ng/L) OR a 20% rise when >20ng/L
❖(i) Patients with chronic TnI elevation and +ve △ require Cardiology Reg review (± 3rd sample at 4hrs); (ii) now asymptomatic patients with chronic elevation and no significant △ (or previously not investigated for this elevation) should have Cardiology SMO review if symptoms were consistent with cardiac ischaemia; or (iii) GP review if TnI was ordered for more vague symptoms as part of general workup.
Ψ Make **Cardiology** E-referral in HCS. Select urgent priority. Write "Cardiology SMO virtual clinic" in 'Reason for referral' box and ask specific question, e.g. "Optimise medical management" or "Cannot perform ETT; are they suitable for CTCA?".
♦ Make **Cardiology-ECG** E-referral in HCS. Select urgent priority. Write "Urgent ETT after ED Chest pain attendance" as reason for referral. Give patient an ETT information sheet (print from hospital health pathways - HHP)
ǂ Investigation not required if (near) normal invasive angiogram within preceding 5 years or CTCA within 2 year or ETT within 1 year.

EDACS (Emergency Department Assessment of Chest Pain Score)

**Age**	☐ 18-45 = **+ 2**<br>☐46-50 = **+ 4**<br>☐51-55 = **+ 6**	☐56-60 = **+ 8**<br>☐61-65 = **+ 10**<br>☐66-70 = **+ 12**	☐71-75 = **+ 14**<br>☐76-80 = **+ 16**<br>☐81-85 = **+ 18**	☐86[+] = **+ 20**	**Sub-score**<br><br>+

Age related risk factors for coronary artery disease (CAD):

☐ **Age 51+**	= 0	(max = 4)	
☐ **Age 18-50** with **NO** known CAD* **AND LESS THAN 3** of the other 5 risk factors	= 0		
☐ **Age 18-50** with **ANY** known CAD* **OR THREE OR MORE** of the other 5 risk factors	= + 4		+

**Other 5 risk factors**	☐ Family history of premature CAD**	☐ Diabetes#<br>☐ Dyslipidaemia#	☐ Hypertension#<br>☐ Current smoker

**Symptoms**	☐ Diaphoresis (in association with pain)❖	= + 3	+
	☐ Pain❖ is pleuritic (sudden worsening with inspiration)	= – 4	-
	☐ Pain❖ radiates to arm/shoulder/neck/jaw	= + 5	+
	☐ Pain❖ reproduced by palpation	= – 6	-
**Gender**	☐ Male = + 6	☐ Female = 0	+

TOTAL SCORE (may be a negative) =

KEY:
* Known CAD = Previous AMI, CABG or PCI
** A first-degree relative with history of an ishaemic cardiac event at <55 yrs if male or <65 yrs if female
\# If patient is on treatment for condition (e.g. statins for dyslipidaemia) select yes.
❖Pain that caused the presentation to hospital

Give all patients ("Adult Cardiac Chest Pain") advice sheet (print from Hospital Health Pathways)
